# Viewpoint Selection for 3D-Games with f-Divergences

**DOI:** 10.3390/e26060464

**Published:** 2024-05-29

**Authors:** Micaela Y. Martin, Mateu Sbert, Miguel Chover

**Affiliations:** 1 Institute of New Image Technologies, Universitat Jaume I, 12071 Castellón, Spain; 2 Institute of Informatics and Applications, University of Girona, 17071 Girona, Spain; mateu.sbert@gmail.com

**Keywords:** viewpoint selection, video games, Kullback–Leibler divergence, χ^2^ divergence, total variation

## Abstract

In this paper, we present a novel approach for the optimal camera selection in video games. The new approach explores the use of information theoretic metrics f-divergences, to measure the correlation between the objects as viewed in camera frustum and the ideal or target view. The f-divergences considered are the Kullback–Leibler divergence or relative entropy, the total variation and the $\chi^{2}$ divergence. Shannon entropy is also used for comparison purposes. The visibility is measured using the differential form factors from the camera to objects and is computed by casting rays with importance sampling Monte Carlo. Our method allows a very fast dynamic selection of the best viewpoints, which can take into account changes in the scene, in the ideal or target view, and in the objectives of the game. Our prototype is implemented in Unity engine, and our results show an efficient selection of the camera and an improved visual quality. The most discriminating results are obtained with the use of Kullback–Leibler divergence.

## 1. Introduction

In the context of 3D virtual scenes in video games, the selection of the best camera position and orientation has not yet received enough attention. Considering the critical importance of visual perception in the development of the game storyline, it is fundamental to develop methods for selecting optimal views that emphasize the most important scene information for the player.

Information theory measures, mainly Shannon entropy, have been widely used in viewpoint selection in robotics, computer graphics and visualization [1,2]. A drawback of these measures is the high associated cost due to their computation with projections.

In this paper we propose the use of f-divergences, exploring the Kullback–Leibler, the total variation, and the $\chi^{2}$ divergence to compute the best viewpoint or camera position in a Unity [3] environment and compare it with the Shannon entropy.

The Kullback–Leibler (K-L) divergence [4] has been introduced as a measure of the best viewpoint of an object in [5,6] and we extend it here to select the best camera in 3D scenes. To measure the visibility of an object, we introduce the camera frustum form factor, extending the work in [7,8]. The form factors of all objects plus the background form factor is a probability distribution that is compared using the K-L divergence with a target distribution, which can be the distribution of relative areas or be weighted with importance values. We also extend the viewpoint entropy to frustum entropy too and show that viewpoint frustum entropy happens when all objects are given importance inversely proportional to their area.

We use the Unity game engine as a development tool because it is one of the most popular and widely used and widely used game engines in the industry, and it offers out-of-the-box ray casting that allows us to compute the form factors in a fast and efficient way.

In this paper we advance on the state of the art along the following novelties:

- We use the f-divergences, and in particular Kullback–Leibler divergence, total variation, and $\chi^{2}$-divergence as a measure of viewpoint in a scene consisting of 3D objects, extending from the use of K-L divergence as a viewpoint measure [5].
- We define the frustum form-factor as a measure of the visibility of an object from a camera, extending the classic form-factor concept used in radiative heat transfer [9], radiosity and global illumination [10,11,12].
- We compute the frustum form factor with a Monte Carlo technique using the built-in ray tracing Unity routines. This allows for smooth computing and integrating the view-point measures in run-time.
- We define a target distribution that can be fine-tuned according to the importance assigned to each object and is extended with a wildcard, the background value which allows to regulate how much background should be visible from the camera.
- The frustum form-factor distribution is then compared, using the f-divergences, with the target distribution.

The rest of the paper is organized as follows. In Section 2 we review the state of the art on viewpoint measures used in robotics and computer graphics and visualization, as well as f-divergences. In Section 3 we present our framework together with the viewpoint divergence measures. Section 4 contains an evaluation of our framework, in Section 5 we discuss the results and in Section 6 we present our conclusions, limitations of our method and future work.

## 2. State of the Art

### 2.1. Viewpoint Selection

The selection of the best point of view in 3D models has been widely investigated in the scientific literature. Plemenos et al. [13] proposed to use projected area and number of polygons seen as the best viewpoint measure. Vazquez et al. [14] proposed the concept of the best viewpoint as the one that maximizes the entropy. The viewpoint entropy measure has proven to be effective in the selection of optimal viewpoints in several applications dealing with 3D environments [15], including robotics [16], and in volumetric data [17].

Bonaventura et al. [6] have proposed a comprehensive classification of attributes, such as area, silhouette, depth, stability and surface curvature to evaluate the quality of a point of view in polygonal models. Some measures included in this category are the number of visible triangles [13], projected area, visibility ratio, viewpoint entropy and Kullback–Leibler viewpoint measure [5]. Silhouette attributes focus on the shape and structure of the object visible from the point of view. Related measures are the length of the silhouette [18], the entropy of the silhouette, the curvature of the silhouette and the extreme of the curvature of the silhouette [19]. Depth attributes focus on the depth of information. Measures in this category are the measure of Stoev and Straßer [20], the maximum depth and the depth distribution. Surface curvature attributes are based on the analysis of the curvature of the surface of the visible object. The stability of the viewpoint is another important aspect to consider when selecting the best viewpoint. Stability attributes evaluate the consistency and continuity between nearby viewpoints. Related measures are instability [21], which is based on the Jensen–Shannon divergence between the projected area distributions and the areas, and visual stability based on depth [22], which uses the normalized compression distance between the depth images of viewpoints.

An important aspect pointed out by Zeng et al. [16] in viewpoint evaluation is the influence of factors such as the occlusion between objects, the different lighting configurations, materials, and textures. These factors can significantly affect the quality of the information collected and, therefore, the choice of the best point of view. Although we acknowledge that this information can be very valuable in gaming contexts, in this paper we currently consider only visibility, colors or textures will be incorporated in the future for a more comprehensive viewpoint evaluation.

In relation to the evaluation of the gain of information in the selection of the point of view, Delmerico et al. [23] propose a comparison of volumetric information metrics for the active reconstruction of 3D objects. Its approach is based on the emission of rays in the 3D space of voxels and the entropy calculation to evaluate the information gain of a particular view.

Zhang and Fei [24] classify viewpoint selection methods into three main categories. The methods based on geometrical information such as the one by Vazquez et al. [14] consider measures such as the area, the projected area, the silhouette and other characteristics of the viewpoint, but they can overlook the structural information of the 3D model. Methods based on visual characteristics focus on visual attributes such as silhouette, curvature [25], and mesh importance [26]. Although these methods are efficient for measuring visual characteristics, they can omit important geometric information in the scene. Finally, the methods based on semantics as proposed in [27,28] evaluate the point of view through the use of semantic segmentation, which considers semantic components of the scene and artificial labels. However, automatic segmentation can be challenging and require manual intervention.

Kullback–Leibler divergence has been used in visualization and computer graphics areas before. Bordoloi et al. [17] have introduced a method for enhancing the effectiveness of volume rendering by guiding users towards informative viewpoints obtained with viewpoint entropy. Kullback–Leibler divergence is employed as a measure of dissimilarity between probability distributions associated with viewpoints. Ruiz et al. [29] introduced a framework for obtaining transfer functions for the volumetric data based on user-provided target distributions. The transfer functions are derived by minimizing the Kullback–Leibler distance between visibility distribution from viewpoints and user-selected target distributions. Lan et al. [30] built a robotic photography system to find the optimal viewpoint of a scene. The system assesses aesthetic composition by comparing, with Kullback–Leibler divergence, the distribution of a current composition with a model or target composition. Smaller Kullback–Leibler divergence values indicate a more aesthetically pleasing composition. Furthermore, Yokomatsu et al. [31] introduce an autonomous indoor drone photographer that searches for a viewpoint in 3D space employing a Gaussian mixture model to represent subjects on its camera screen. Using variational Bayes clustering for four or more subjects, it evaluates the composition through Kullback–Leibler divergence against a user-defined reference based on user-set composition rules. To the best of our knowledge, although K-L divergence has been used for various applications, it has not been used for viewpoint selection in open environments and multi-object scenes, neither total variation nor $\chi^{2}$-divergence have been used in viewpoint selection, in general.

In the field of video games, the selection of the best viewpoint for virtual scenes has received less attention compared to robotics. There are few studies that specifically address this topic in video games, especially those that work with virtual scenes in real time. An example of this is the work conducted by Galvane [32], where he proposes a system based on the Reynolds steering behavior model to control and coordinate a collection of autonomous camera agents that move in dynamic 3D environments with the objective of filming events of multiple scales. In addition, Galvane proposes an approach based on the importance of cinematographic reproduction in games, taking advantage of the narrative and geometric information to automatically calculate the trajectories and the planning of the camera in interactive time. The best camera viewpoints are selected based on a function that takes into account symbolic projection, narrative importance, narrative relevance, and visual quality. Virtual camera rails are then created to guide camera movements throughout the scene, and camera movements are calculated, optimizing the trajectory to achieve smooth transitions.

Another approach proposed by Lino and Christie [33] is the use of a theoretical surface model that efficiently generates a variety of viewpoints corresponding to the exact on-screen composition of two or three objectives. These approaches, based on algebraic models, offer fast and efficient solutions for the automatic calculation of points of view, although they tend to be limited to a small number of objectives and may not address cinematographic problems such as obstruction or occlusion.

The use of neural networks has also been proposed for the selection of viewpoints in 3D environments. Zhang et al. [34] presented an optimization strategy for computing high-quality virtual viewpoints for aesthetic images by combining a multi-branch CNN and a viewpoint correction method, integrating visual perception with the calculation of geometric information. Furthermore, deep learning has been utilized to reconstruct the 3D pose from the image obtained in the video by Kiciroglu et al. [35]. An algorithm was created that, based on the camera position, calculates the uncertainty and generates a set of future camera positions, taking into account that the scene is unknown. The authors have then used neural networks to return the 3D human pose from monocular images.

Hartwig et al. [36] introduced a neural view quality measure aligned with human preferences. The study demonstrated that this measure generalized not only to models unseen during training but also to unseen model categories.

### 2.2. f-Divergences

f-divergences generalize the Kullback–Leibler divergence between two distributions [37,38,39]. Given p,q distributions ${\left\{p_{i}\right\}}_{i=1}^{n},{\left\{q_{i}\right\}}_{i=1}^{n}$, $\forall i:p_{i},q_{i}\geq 0$, f-divergence $D_{f}\left(p,q\right)$ is defined as:

$$D_{f}\left(p,q\right)=\sum_{i=1}^{n}q_{i}f\left(p_{i}/q_{i}\right),$$

where f(x) is a real-valued convex function such that f(1)=0. By definition 0f(0/0)=0, and for a>0, $0f\left(a/0\right)=\lim_{t^{+}\to 0}tf\left(\frac{a}{t}\right)$.

$D_{f}\left(p,q\right)$ holds the properties:

- $D_{f}\left(p,q\right)\geq 0$, and $p\equiv q\Rightarrow D_{f}\left(p,q\right)=0$ ($D_{f}\left(p,q\right)=0$⇒p≡q for f(x) strictly convex).
- $D_{f}\left(p,q\right)$ is convex in both *p* and *q*.
- $D_{f}\left(p,q\right)\equiv D_{f(x)+c(x-1)}\left(p,q\right)$.
- Given a transform *T*, $D_{f}\left(p,q\right)\geq D_{f}\left(pT,qT\right)=0$ (data processing inequality-DPI). In particular, *T* can be any clustering of indexes.

Well known examples of f-divergences are K-L divergence or relative entropy, with f(x)=xlogx, $\mathrm{K-L}\left(p,q\right)=\sum_{i=1}^{n}p_{i}\log\frac{p_{i}}{q_{i}}$, total variation, with f(x)=1/2|x−1|, $TV\left(p,q\right)=\frac{1}{2}\sum_{i=1}^{n}\left|p_{i}-q_{i}\right|$, and which is the only f-divergence that is also a Euclidian distance, squared-Hellinger distance, with $f\left(x\right)={\left(\sqrt{x}-1\right)}^{2}$, $H^{2}\left(p,q\right)=\sum_{i=1}^{n}{\left(\sqrt{p_{i}}-\sqrt{q_{i}}\right)}^{2}$ and $\chi^{2}$-divergence, with $f\left(x\right)={\left(x-1\right)}^{2}$, $\chi^{2}\left(p,q\right)=\sum_{i=1}^{n}\frac{{\left(p_{i}-q_{i}\right)}^{2}}{q_{i}}$. Another example is the one-parameter family of f-divergences, the Tsallis divergences, with α>0,

$$T_{\alpha }\left(p,q\right)=\frac{1}{1-\alpha }\left(\sum_{i=1}^{n}\frac{p_{i}^{\alpha }}{q_{i}^{\alpha -1}}-1\right),$$

which includes for α=2 the $\chi^{2}$ divergence, for α=1/2 the squared Hellinger distance, and for α=1 by continuity the K-L divergence [40].

Observe that Shannon entropy, $H\left(p\right)=-\sum_{i=1}^{n}p_{i}\log p_{i}$ is related to the Kullback–Leibler divergence when *q* distribution is uniform, i.e., when $q={\left\{\frac{1}{n}\right\}}_{i=1}^{n}$,

$$\mathrm{K-L}\left(p,\left\{1/n\right\}\right)=\sum_{i=1}^{n}p_{i}\log\frac{p_{i}}{1/n}=\log n\sum_{i=1}^{n}p_{i}-\sum_{i=1}^{n}p_{i}\log p_{i}=\log n-H\left(p\right).$$

## 3. Proposed Method

The intuition behind our method is to measure the visibility of the objects from the camera and compare the distribution of visibility to the distribution of the areas using f-divergences. In this aspect, the more informative view would be when each object is visible proportionally to its area. The lower the divergence value, the more proportional are visibility and relative area distributions.

### 3.1. Visibility

In the field of computer graphics and video game development, two fundamental concepts related to visual perception and projection are the Field of View (FOV) and the Frustum. The Field of View refers to the angular range of the observable scene or the visual range of a camera in a three-dimensional environment. A wider FOV provides a more extensive peripheral vision, while a narrower FOV focuses on a smaller area with greater detail. On the other hand, the Frustum represents the shape of a truncated pyramid.

In previous work by Rigau et al. [41], the visibility of a point in a 3D scene was studied based on information theoretic criteria. This work proposed to use the Kullback–Leibler divergence between the solid angles projected by the objects and the unoccluded projections as a measure of the viewpoint. Also, Sbert et al. [5] proposed as a viewpoint measure of an object the K-L divergence between the projected areas of the triangles of the object mesh and the true areas. We extend these ideas to consider the visibility measured by the form factors from the viewpoint [7,8], constrained to the camera frustum, and we consider two additional divergence measures in addition to K-L divergence.

**Figure 1:**
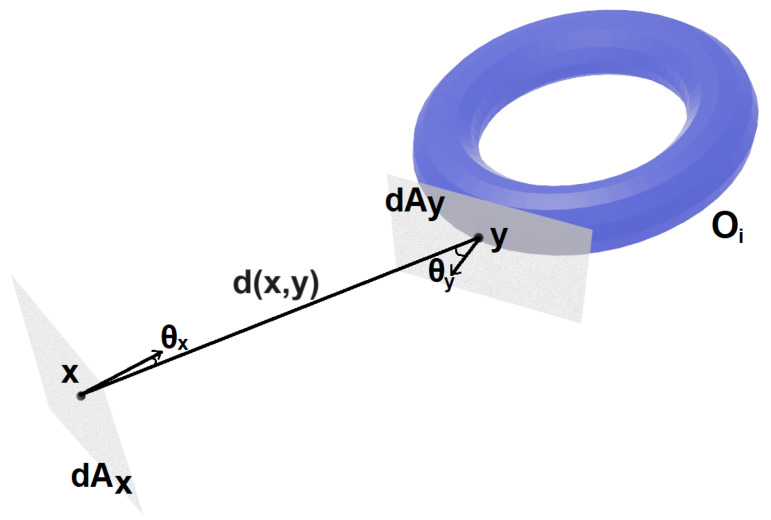
Notation used for differential form-factor, *x* is the camera position, $dA_{x}$ is on the camera plane, *y* is a point on the surface of object $o_{i}$ at distance d(x,y) from *x*, $dA_{y}$ is on the tangent plane at *y*, $\theta_{x}$ and $\theta_{y}$ are the angles between the normals at *x* and *y* and the line joining *x* and *y* respectively.

Given a scene $S\subset R^{3}$, and *O* the set of objects $\left\{o_{i}\right\}$ in the scene. We define $A_{i}$ as the area of object $o_{i}$, $A_{T}$ as the total area of the objects in the scene plus background area, and $a_{i}=\frac{A_{i}}{A_{T}}$ as the relative area of object $o_{i}$. Given the position x∈S of the camera, we consider $dA_{x}$ in the normal plane to the camera direction. Given a point *y* in the surface of an object in *O*, $dA_{y}$ is in the tangent plane at *y*, $\theta_{y}$ is the angle between the normal to $dA_{y}$ with the line that joins *x* and *y*, $\theta_{x}$ is the angle with the normal at $dA_{x}$, and d(x,y) the distance between *x* and *y* (see Figure 1).

### 3.2. Hemisphere Form-Factors

$F(dA_{x},dA_{y})$ is the differential form factor (or measure of the visibility) between differential areas $dA_{x},dA_{y}$ at point *x*. It forms a continuous probability distribution, i.e.,

$$\int_{y\in O}F\left(dA_{x},dA_{y}\right)dA_{y}=1,$$

where:

(1) $$F\left(dA_{x},dA_{y}\right)dA_{y}=\frac{\cos\theta_{x}\cos\theta_{y}}{\pi d{\left(x,y\right)}^{2}}v\left(x,dA_{y}\right)dA_{y},$$

and $v(x,dA_{y})$ is a binary visibility function (equal to 1 if *x* and *y* are mutually visible and 0 otherwise). If dω is the area subtended by $dA_{y}$,

(2) $$F\left(dA_{x},dA_{y}\right)d\omega =\frac{1}{\pi }v\left(x,dA_{y}\right)\cos\theta_{x}d\omega ,$$

taking into account that $d\omega =\frac{\cos\theta_{x}\cos\theta_{y}}{d{\left(x,y\right)}^{2}}dA_{y}$, and the π factor is the normalization constant as the integral over the hemisphere is $\int_{\Omega /2}\cos\theta_{x}d\omega =\pi$. The form factor for object *i* around the hemisphere centered in $dA_{x}$ is then defined as

(3) $$F\left(dA_{x},o_{i}\right)=\int_{\Omega /2}\frac{1}{\pi }v\left(x,o_{i}\left(\omega \right)\right)\cos\theta_{x}d\omega =\int_{\Omega_{i}}\frac{1}{\pi }\cos\theta_{x}d\omega ,$$

where $v(x,o_{i}\left(\omega \right))$ is 1 or 0 depending on whether object $o_{i}$ is visible or not from direction ω, and $\Omega_{i}$ is the solid angle over the hemisphere around $dA_{x}$ from which the object *i* is visible. If $\Omega_{b}=\Omega /2-\sum_{i}\Omega_{i}$ is the solid angle projected by the background, we can write as only one object (or the background) is visible in a given direction ω

(4) $$\begin{array}{ccc} & & \int_{\Omega /2}\frac{1}{\pi }\cos\theta_{x}d\omega =1=\sum_{i}\int_{\Omega_{i}}\frac{1}{\pi }\cos\theta_{x}d\omega +\int_{\Omega_{b}}\frac{1}{\pi }\cos\theta_{x}d\omega \\ & = & \sum_{i}F\left(dA_{x},o_{i}\right)+F\left(dA_{x},o_{b}\right), \end{array}$$

where $F(dA_{x},o_{b})$ is the background form-factor. However, the whole hemisphere is not visible through a virtual camera, thus in the next section, we restrict the visibility to the camera frustum.

### 3.3. Frustum Form-Factors and f-Divergence Frustum Viewpoint Measures

Let us consider the visibility restricted to a frustum fr that subtends a solid angle $\Omega_{fr}$ around $dA_{x}$. The normalization constant $k_{fr}$ for fr form-factors would be

$$k_{fr}=\int_{\Omega_{fr}}\cos\theta_{x}d\omega$$

and can be computed by importance sampling Monte Carlo integration [42]. We can use for instance *N* rays distributed around the hemisphere with probability density function (pdf) $p\left(\omega \right)=1/\pi \cos\theta_{x}d\omega$, in which case $k_{fr}\approx \pi N_{fr}/N$, where $N_{fr}$ is the number of rays crossing the frustum. We define the frustum form factor as

(5) $$F\left(dA_{x},o_{i}\right)=\int_{\Omega_{i}\cap \Omega_{fr}}1/k_{fr}\cos\theta_{x}d\omega ,$$

Equation (5) can be computed efficiently by Monte Carlo by casting rays distributed according to $p\left(\omega \right)=1/\pi \cos\theta_{x_{i}}$ and simply counting the fraction of rays within the frustum and hitting the object $o_{i}$ (see Appendix A). This can be conducted for all objects $\left\{o_{i}\right\}$ at the same time. Let a background object be $o_{b}$, for instance, a background hemisphere, then we have that

$$\sum_{i=1}^{n}F\left(dA_{x},o_{i}\right)+F\left(dA_{x},o_{b}\right)=1.$$

Let us now consider the fraction of the total area corresponding to each object, say $a_{i}$. For the background hemisphere, we have $a_{b}$. Indeed

$$\sum_{i=1}^{n}a_{i}+a_{b}=1.$$

Then we can consider the f-divergence measures between the form-factor distribution and the relative area distribution.

#### 3.3.1. Kullback–Leibler Divergence

We will consider first the Kullback–Leibler divergence between the two distributions,

(6) $$\mathrm{K-L}\left(dA_{x},\left\{o_{i}\right\}\right)=\sum_{i=1}^{n}F\left(dA_{x},o_{i}\right)\log\frac{F(dA_{x},o_{i})}{a_{i}}+F\left(dA_{x},o_{b}\right)\log\frac{F(dA_{x},o_{b})}{a_{b}},$$

where if, for some *i*, $F(dA_{x},o_{i})=0$, we take continuity $F\left(dA_{x},o_{i}\right)\log\frac{F(dA_{x},o_{i})}{a_{i}}=0$. And using the hit count, where $N_{i}$ is the number of hits on object *i*, and $N_{b}$ on the background, the frustum form factor is approximated by

(7) $$F\left(dA_{x},o_{i}\right)\approx \frac{N_{i}}{N_{fr}},$$

and thus the Kullback–Leibler divergence is approximated by

(8) $$\mathrm{K-L}\left(dA_{x},\left\{o_{i}\right\}\right)\approx \sum_{i=1}^{n}\frac{N_{i}}{N_{fr}}\log\frac{\frac{N_{i}}{N_{fr}}}{a_{i}}+\frac{N_{b}}{N_{fr}}\log\frac{\frac{N_{b}}{N_{fr}}}{a_{b}}.$$

Observe that $a_{i}=A_{i}/\left(A_{T}+A_{b}\right)$, and $a_{b}=A_{b}/\left(A_{T}+A_{b}\right)$, and $A_{T}=\sum_{i}A_{i}$.

Now, taking the Kullback–Leibler divergence as a viewpoint measure has one problem: it does not penalize not-seen objects, on the contrary, the corresponding term in the sum is 0. Let us then consider two alternatives.

#### 3.3.2. Total Variation and $\chi^{2}$-Divergence Frustum Viewpoint Measures

Now, using the total deviation as a frustum viewpoint measure, we obtain

(9) $$TV\left(dA_{x},\left\{o_{i}\right\}\right)\approx 1/2(\sum_{i=1}^{n}|\frac{N_{i}}{N_{fr}}-a_{i}|+|\frac{N_{b}}{N_{fr}}-a_{b}|).$$

And using $\chi^{2}$-divergence,

(10) $$\chi^{2}\left(dA_{x},\left\{o_{i}\right\}\right)\approx \sum_{i=1}^{n}\frac{{\left(\frac{N_{i}}{N_{fr}}-a_{i}\right)}^{2}}{a_{i}}+\frac{{\left(\frac{N_{b}}{N_{fr}}-a_{b}\right)}^{2}}{a_{b}}.$$

Observe that in both measures an object $o_{i}$ non-visible adds the same amount $a_{i}$. This is the main difference with respect to the K-L measure, where the race of a non-visible object disappears.

Another approach, proposed by Lino and Christie [33], is the use of a theoretical surface model that efficiently generates a variety of viewpoints corresponding to the exact on-screen composition of two or three objectives. These approaches based on algebraic models offer fast and efficient solutions for the automatic calculation of points of view, although they tend to be limited to a small number of objectives and may not address cinematographic problems such as obstruction or occlusion.

### 3.4. Background Issues and Importance

Suppose we are in an open scene with no background surface(s) to consider. How do we deal with this case? On the one hand, we consider the rays missing the objects $\left\{o_{i}\right\}$ as hitting the background, and we count them as $N_{b}$. Now, instead of considering some fictitious background surface as a hemisphere enveloping the objects we can decide a priori how much background we want to see in our frustum, and simply set $a_{b}$ as a proportion of $\sum_{i=1}^{n}a_{i}$. A small $\frac{a_{b}}{\sum_{i}a_{i}}$ value means that our viewpoint measure will favor a small background proportion of the frustum, while a big one will mean the reverse. This can be extended to any object in $\left\{o_{i}\right\}$. It can be formalized, in a similar way to [21], by defining importance non zero values $\left\{p_{i}\right\}$ for objects and background $p_{b}$ and considering the new pseudo-area distribution $a_{i}^{\prime }=\frac{a_{i}p_{i}}{\sum_{k}a_{k}p_{k}+a_{b}p_{b}}$, $a_{b}^{\prime }=\frac{a_{b}p_{b}}{\sum_{k}a_{k}p_{k}+a_{b}p_{b}}$.

### 3.5. Total Surface vs. Visible Surface

We have considered in the previous sections $\left\{a_{i}\right\}$ as the relative surface area of objects $\left\{o_{i}\right\}$. But for each object, we could have considered the visible area $\left\{a_{i}^{\prime }\right\}$, where evidently ∀i, $a_{i}^{\prime }\leq a_{i}$. This would make sense if objects have an important share of hidden parts. The $\left\{a_{i}^{\prime }\right\}$ could be computed in a preprocess, using for instance global uniformly distributed lines [8]. A similar discussion between visible and total areas can be found in [21].

### 3.6. Particular Cases with Kullback–Leibler Divergence

Let us suppose that only a single object $o_{i}$ is visible through a particular frustum. Then, $N_{i}/N_{fr}=1$, and the K-L divergence would be $-\log a_{i}$. Observe that this value is independent of how near the object is, as far as it covers the whole frustum. For instance, suppose $a_{i}=1/32$, then the K-L divergence would be equal to 5. The same would happen if only the background is visible, the K-L value would be $-\log a_{b}$. For instance, giving low importance to the background, say $a_{b}=1/64$, the K-L value would be equal to 6, while giving it much higher importance, say $a_{b}=1/2$, the K-L value would be equal to 1.

### 3.7. K-L-Divergence Frustum Viewpoint Measure versus Frustum Viewpoint Entropy

Analog to the classic viewpoint entropy [14], we define frustum viewpoint entropy as

(11) $$\begin{array}{c} VE\left(dA_{x},\left\{o_{i}\right\}\right)=-\left(\sum_{i=1}^{n}F\left(dA_{x},o_{i}\right)\log F(dA_{x},o_{i})+F\left(dA_{x},o_{b}\right)\log F(dA_{x},o_{b})\right), \end{array}$$

with *n* as the number of objects $\left\{o_{i}\right\}$. The best view according to this measure would be the one with the highest value, which corresponds to all objects and backgrounds seen equally, all form factors are equal, independent of their relative areas.

Observe that if we take the K-L divergence frustum viewpoint for all objects including background having the same relative area, or identically if we take the importance of each object including a background as inversely proportional to their area, the relative area for all objects and background is now $\frac{1}{n+1}$, thus we have

(12) $$\begin{array}{ccc} \mathrm{K-L}(dA_{x},\left\{o_{i}\right\}) & = & \sum_{i=1}^{n}F\left(dA_{x},o_{i}\right)\log\frac{F(dA_{x},o_{i})}{\frac{1}{n+1}}+F\left(dA_{x},o_{b}\right)\log\frac{F(dA_{x},o_{b})}{\frac{1}{n+1}}\\ & = & \sum_{i=1}^{n}F\left(dA_{x},o_{i}\right)\log F(dA_{x},o_{i})+F\left(dA_{x},o_{b}\right)\log F(dA_{x},o_{b})\\ & + & \log\left(n+1\right)(\sum_{i=1}^{n}F\left(dA_{x},o_{i}\right)+F\left(dA_{x},o_{b}\right))\\ & = & -VE\left(dA_{x},\left\{o_{i}\right\}\right)+\log\left(n+1\right). \end{array}$$

This is, in that particular case one can indistinctly use K-L divergence or entropy measure, just now, the best views with K-L measure would be the ones with the lowest value, and for entropy the ones with the highest value.

### 3.8. TV Changes Smoothly

The fact that TV is a Euclidean distance allows us to bound the increment of TV measure when we consider another frustum or when we change the relative areas (for instance background area). Suppose we change from a frustum with form-factors $\left\{F_{i}\right\}$ to another frustum with form-factors $\left\{F_{i}^{\prime }\right\}$ (or the same frustum but objects have moved position). Suppose also that we change areas from $\left\{a_{i}\right\}$ to $\left\{a_{i}^{\prime }\right\}$ (for instance, changing background area). Then, we can bound the change in TV measure. Effectively, using the symmetric and triangular inequality properties of a Euclidean distance we can state the following inequalities,

$$TV\left(F,a^{\prime }\right)\geq TV\left(F,a\right)-TV\left(a,a^{\prime }\right),$$

$$TV\left(F,a^{\prime }\right)\leq TV\left(F,a\right)+TV\left(a,a^{\prime }\right),$$

$$TV\left(F^{\prime },a\right)\geq TV\left(F,a\right)-TV\left(F,F^{\prime }\right),$$

$$TV\left(F^{\prime },a\right)\leq TV\left(F,a\right)+TV\left(F,F^{\prime }\right).$$

Observe that these bounds imply that a small change in the form factors or in the area implies a small change in the TV viewpoint measure, i.e., it changes smoothly. This is not always the case with K-L and $\chi^{2}$ measures.

### 3.9. Rays vs. Projection

Projection has been used in the past to compute viewpoint measures of a 3D object or to simplify its mesh based on a viewpoint measure. Could we equally use in the context of this paper projection instead of casting rays to compute the f-divergence frustum viewpoint measure? Let us remember first that we base our viewpoint measure on the form factor measuring the visibility. Before switching to ray casting, form factors were computed via projecting on the five faces of a hemicube [43], where the pixels of the faces had unequal weights. This gave an approximation to the actual value of the form factor. Implementing hemicube in a game engine would be tricky. Sillion and Puech [44] computed form factors by substituting the five projections with a single projection, but objects near the horizon were missed. On the other hand, ray casting can compute the actual values, up to the statistical error, and also each ray can be computed independently, as the Monte Carlo method is by nature parallelizable. In addition, it is very simple to program and game engines support it in real time.

### 3.10. Implementation

The proposed method has been implemented in the Unity game engine making use of its ray-casting routines.

## 4. Evaluation

**Figure 2:**
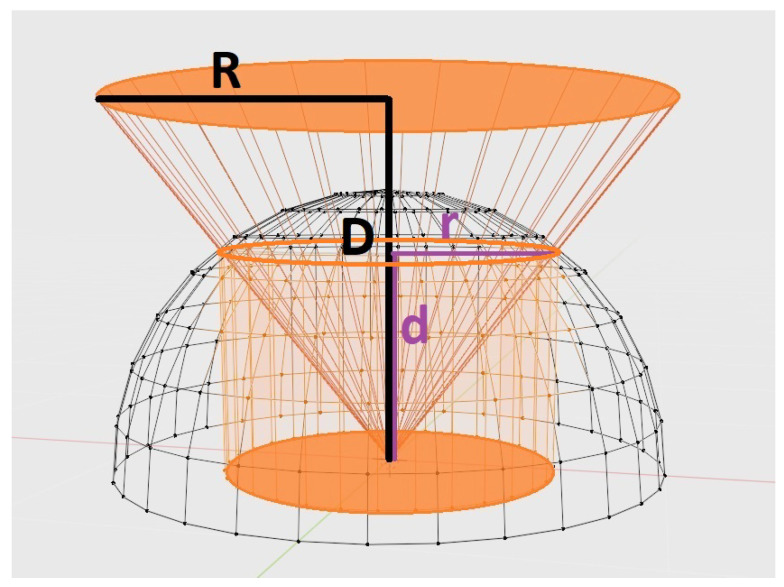
To check the correct distribution of cast lines, we compare the experimental result with the analytically computed form factor corresponding to a disk (in orange color) with radius R at distance D from the camera and orthogonal to its plane. The form factor corresponds to the area of the projected circle, $\pi r^{2}$, divided by the area of the disk with radius 1, π, this is $\frac{\pi r^{2}}{\pi }=r^{2}$. By similarity of the triangles, $k=\frac{d}{r}=\frac{D}{R}$. As $d^{2}+r^{2}=1$, we can easily find that $r^{2}=\frac{1}{k^{2}+1}$, and thus the form factor is $\frac{1}{k^{2}+1}$.

To check the correct computation of the form factor, we devised a configuration where the form factor can be computed analytically, see Figure 2. The results are shown in Table 1. As a form-factor $F_{i}$ computed with random rays corresponds to a binary hit or miss distribution, its variance or expected quadratic error is $\frac{F_{i}\left(1-F_{i}\right)}{N_{rays}}$, and thus the expected error is $\sqrt{\frac{F_{i}\left(1-F_{i}\right)}{N_{rays}}}$, which is compared in the table with the experimental error.

**Figure 3:**
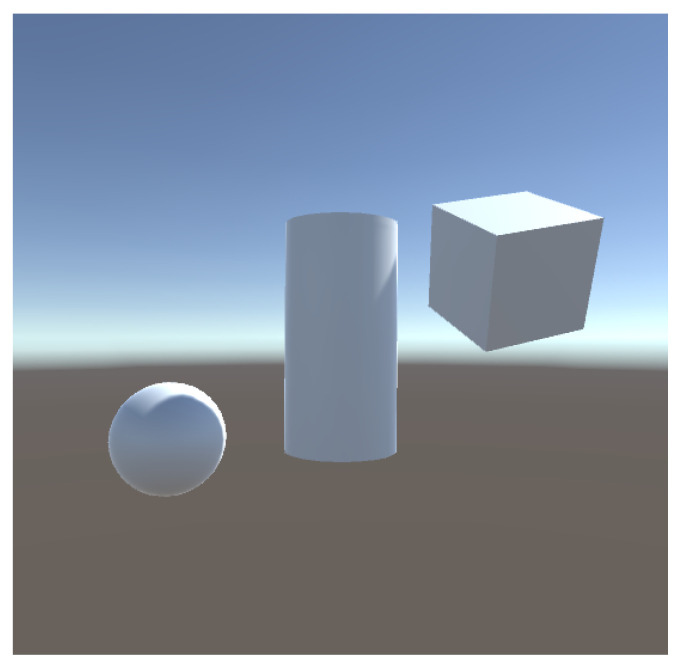
Scene used for the measurements in Table 2.

To study the error in the three viewpoint measures considered we compute them for Figure 3 with varying number of rays. As we can see in Table 2, by casting 100,000 rays the change in the results between the different iterations is less than 0.01, thus a difference of 0.02 will be considered significant to compare two viewpoints.

**Figure 4:**
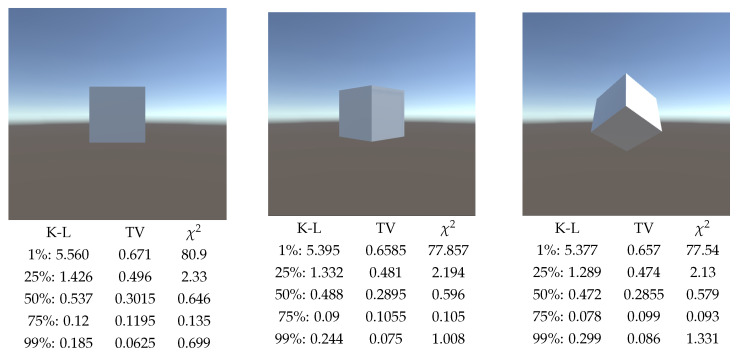
Comparison of measures (TV: Total Variation, K-L: Kullback–Leibler, and $\chi^{2}$) by rotating a single cube, and computed with 100,000 rays. Percentages are the relative area assigned to background.

Then, given that the background area was added as a variable due to the fact that the background has no area in Unity, different tests were carried out to evaluate if there is a suitable background area percentage in general. The first scene considered was a single cube, rotated to obtain three different views of it, see Figure 4, and considering the background area values of 1%, 25%, 50%, 75% and 99% of the total area.

We note that if we assign to the background a relative area equal to its experimental form factor for each view, the measures would give the same result while seeing the objects from far or near. Thus, to avoid this, the relative area of the background should be kept fixed at a relative percentage of the total area.

We observe that for background areas of 1%, 25%, 50%, and 75%, the value of the three measures decreases as the number of visible faces of the cube increases. The only case where the measure increases when the number of visible faces of the cube increases corresponds to the background 99% of the total area, which makes sense considering that increasing the number of visible faces decreases the area occupied by the background.

**Figure 5:**
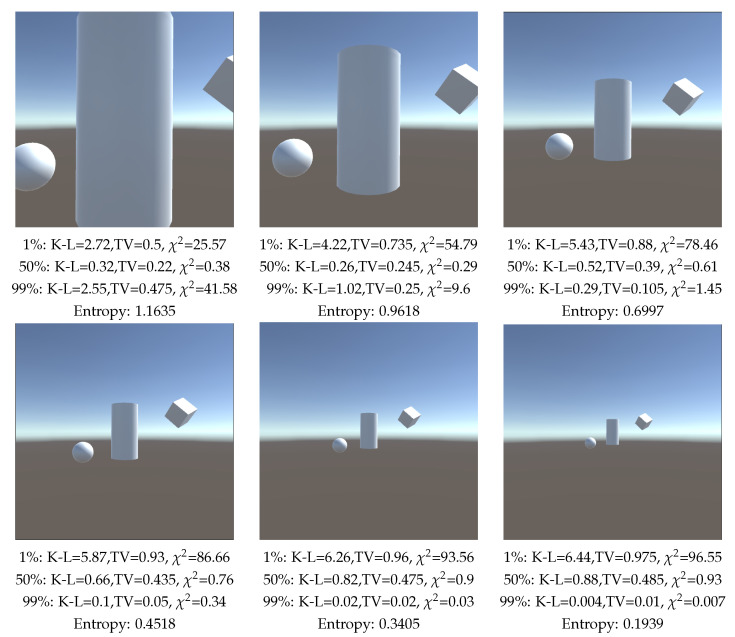
Comparison of measures (TV: Total Variation, K-L: Kullback–Leibler, $\chi^{2}$ and Shannon entropy) when zooming out the camera, and computed with 100,000 rays. Percentages are the relative area assigned to background.

Then, a scene composed of a cube, a cylinder and a sphere, Figure 5, is analyzed, placed in the center of the scene and zoomed in and out, and for values of the background area of 1%, 50%, and 99% of the total area. We also compute viewpoint entropy. When analyzing the values assigned to the background, we notice that by assigning to it a low value (1%), the lowest measure values will be when the more percentage of the FOV of the camera is occupied by objects, while zooming in the lowest value will be the ones in Figure 5 upper left image for all the measures. Analyzing the case where the background value is 99%, the measurements will decrease by zooming out the camera, thus, the measurement is minimal in Figure 5 lower right. Concerning the viewpoint entropy measure, remember that its behavior is inverse to the one of the K-L measures; the higher value would correspond to a better view. It decreases when zooming out, and increases with zooming in, which is correct. However, in Figure 5 upper left, when we zoom in on the cylinder object, entropy is still growing, which is not the expected behavior for a good view, as it is clearly a worse view than Figure 5 upper center. On the other hand, by assigning to the background an area of 50% of the total area, K-L and $\chi^{2}$ measures achieve a minimum in Figure 5 upper center, which we consider to be the best view. The TV measure decreases too when zooming in, but its minimum is not achieved in Figure 5 upper center but in Figure 5 upper left.

**Figure 6:**
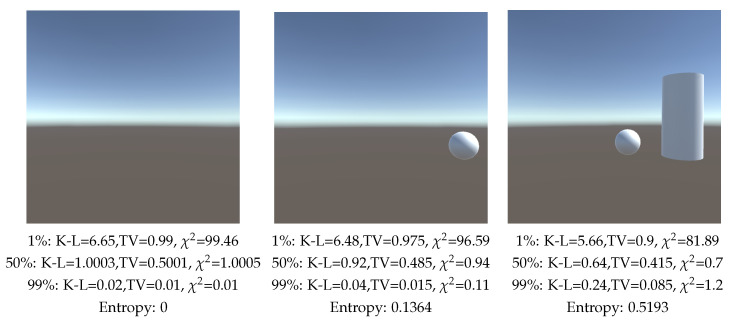

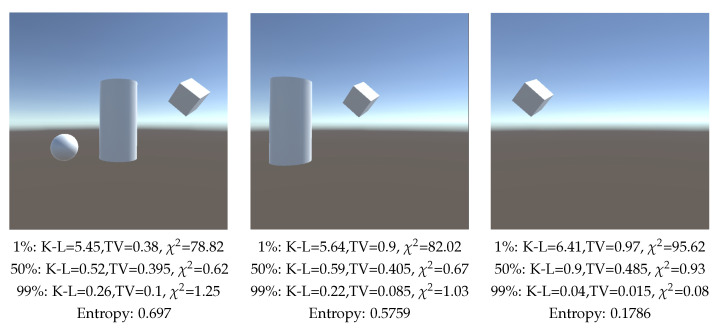
Comparison of measures (TV: Total Variation, K-L: Kullback–Leibler, $\chi^{2}$ and Shannon entropy) when rotating the camera around a vertical axis, and computed with 100,000 rays. Percentages are the relative area assigned to background.

As a third example, as seen in Figure 6, the camera was rotated around a vertical axis obtaining different views of a scene composed of a cube, a cylinder and a sphere. For a 1% background value as well as for 50%, the lowest value for the three divergences happened when the three objects were visible, while for a background value of 99% the lowest value for the divergences happened when no object was visible. For Shannon entropy, the highest value is found when all objects are visible while the lowest value is when no object is visible. Observe that the behavior of the entropy measure is inverse to the one of the K-L divergence. Remember that entropy does not take into account the area of objects and background, and behaves inversely as the K-L divergence when all objects, including background, have the same relative area, thus background value does not play any role in the entropy measure computation. In this example, the entropy measure gives results coherent with our expectation of a good view.

From our results, we infer that assigning the background importance of 50% gives good results.

**Figure 7:**
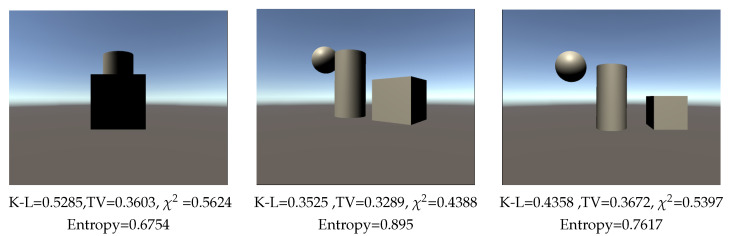
Comparison of measures (TV: Total Variation, K-L: Kullback–Leibler, and $\chi^{2}$) for occlusion, and computed with 100,000 rays. Background area = 50%.

To test the measures in the presence of occlusion, we employed a scene composed of a cylinder, a sphere, and a cube. The scene was observed from lateral (Figure 7 left), diagonal (Figure 7 center), and frontal (Figure 7 right) views while maintaining a constant distance to the cylinder (Figure 7). The results show that K-L identifies as the best viewpoint the diagonal view, where a larger area is visible and there is minimal occlusion between objects. Additionally, it designates the lateral view as the least favorable, where greater occlusion among objects is present. Similarly, $\chi^{2}$ and Shannon entropy perform well; TV, while identifying the diagonal view as the best, does not distinguish between the lateral and frontal ones.

**Figure 8:**
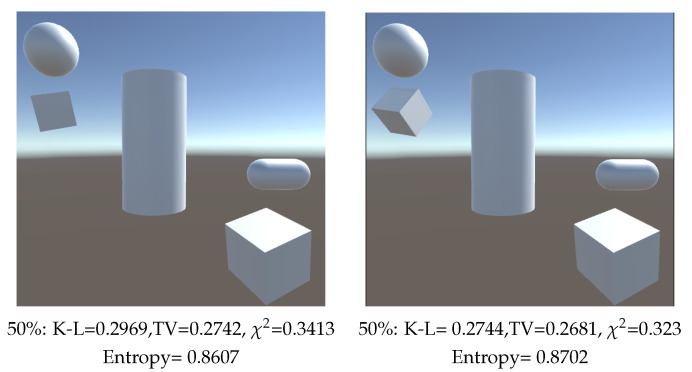
Comparison of measures (Entropy, TV: Total Variation, K-L: Kullback–Leibler and $\chi^{2}$) when rotating one single object, and computed with 100,000 rays. Percentages are the relative area assigned to background.

Finally, in Figure 8 we find two scenes with five objects each, the scenes only differ in the rotation of one cube. While K-L divergence clearly appreciates an improvement in the second view (Figure 8 right), TV (see Section 3.8) and $\chi^{2}$ divergences and Shannon Entropy improvement falls within the threshold we established of 0.02.

### 4.1. Validation in a Video Game Environment

**Figure 9:**
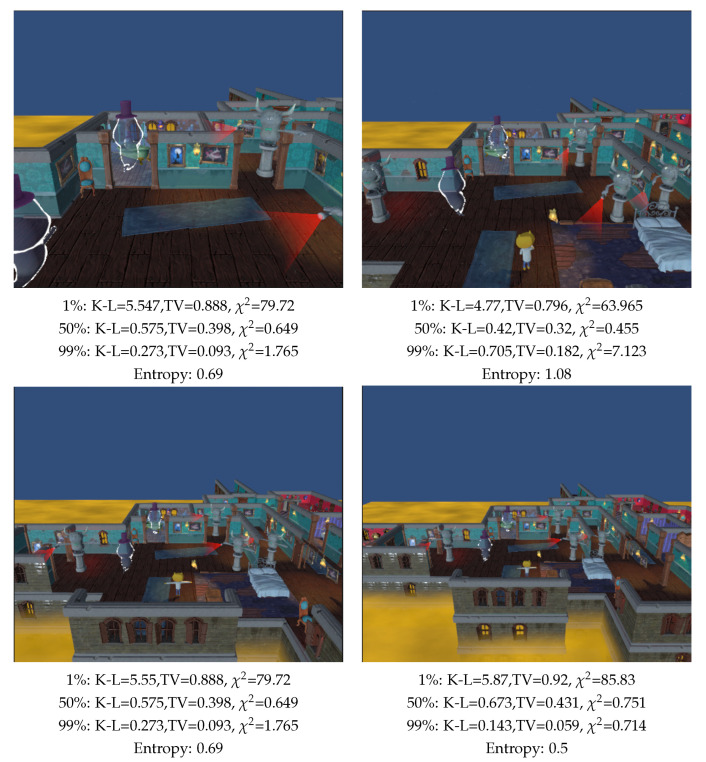
Comparison of measures (TV: Total Variation, K-L: Kullback–Leibler, $\chi^{2}$ and Shannon entropy) when zooming out the camera in a videogame scene, and computed with 100,000 rays. The main character is a kitty with a yellow color head, enemies are grey color ghosts and grey color gargoyles with a red torch. Percentages are the relative area assigned to the background.

**Figure 10:**
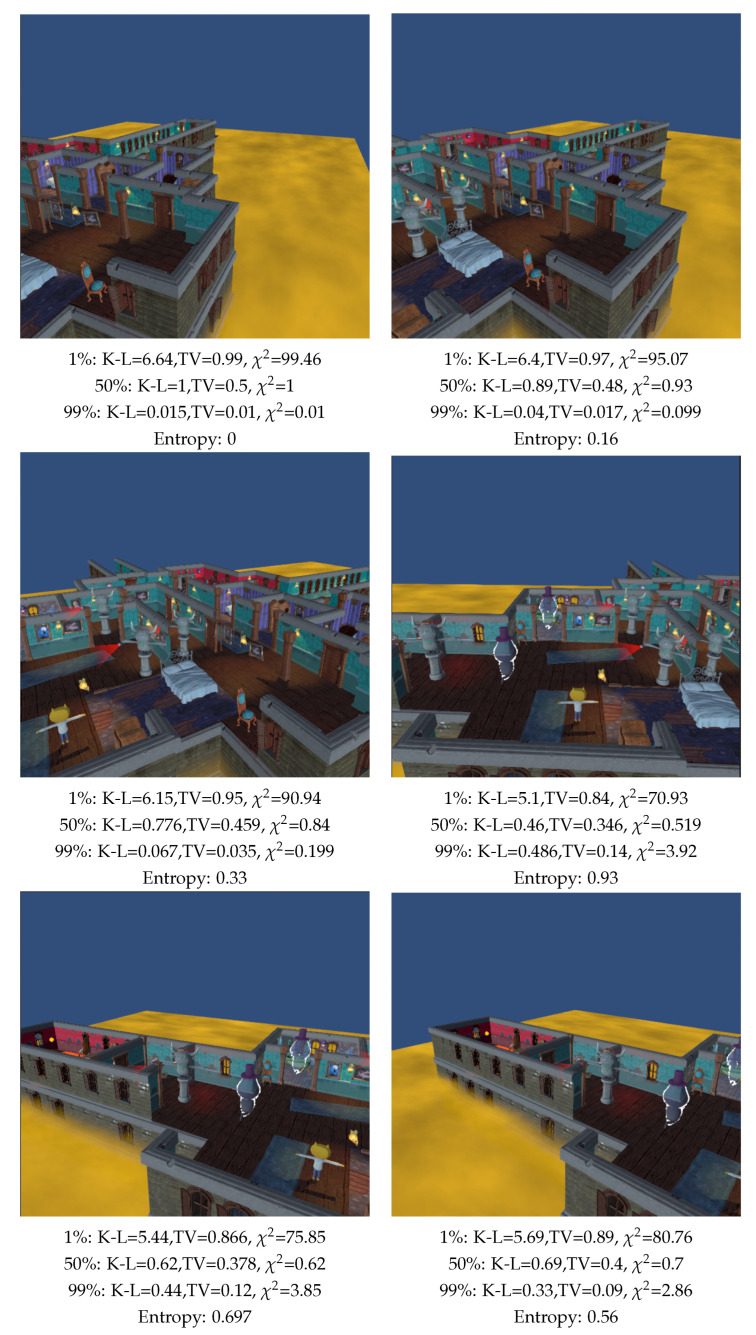
Comparison of measures (TV: Total Variation, K-L: Kullback–Leibler, $\chi^{2}$ and Shannon entropy) when rotating the camera around a vertical axis in a video game scene, and computed with 100,000 rays. Main character is a kitty with yellow color head, enemies are grey color ghosts and grey color gargoyles with a red torch. Percentages are the relative area assigned to background.

To validate the implementation in a video game, we tested the method in the John Lemmon Unity game [45]. The first two evaluated scenes, depicted in Figure 9 and Figure 10, consist of the main character (a yellow-headed kitty), six enemies (three grey gargoyles holding a red lantern in their hand and three grey ghosts with a purple hat), and the background. Similar to the analysis in Figure 5, we initially examined what happens when the camera is zoomed in and out of the scene for background area values of 1%, 50%, and 99% of the total area. As before, assigning a low value (1%) to the background area results in lower measurement values when a higher percentage of the camera’s field of view is occupied by objects. In this case, it corresponds to the upper-right image in Figure 9, while the highest value occurs when the background occupies the largest percentage, as seen in the lower-right image in Figure 9. Analyzing the case where the background value is 99%, the measurements decrease as the camera moves away from the objects. Consequently, the measurements are minimal in the lower-right image in Figure 9. In this scenario, entropy selects the upper-right image in Figure 9 as the best view, likely because the object areas are similar. This view also emerges as the optimal one when the background area comprises 50% of the total area for all three studied measurements. As for Figure 10, the best view is the central right one by giving the background 1% and 50% of the total area, a view that also coincides with the best one for entropy. On the other hand, when giving the background an area of 99% of the total area, the best view is the top left where no objects are observed. These results are consistent with the previous results, highlighting that views can be considered good when the background area represents 50% of the total area. With this in mind, we analyze the following example.

**Figure 11:**
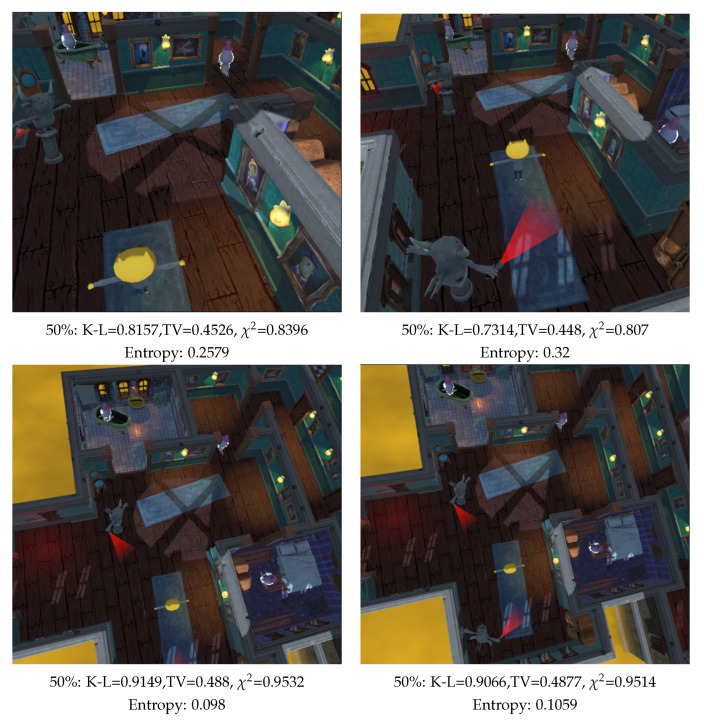
Comparison of measures in a video game (TV: Total Variation, K-L: Kullback–Leibler, $\chi^{2}$ and Shannon entropy, percentages are the relative area assigned to background), and computed with 100,000 rays. The main character is a kitty with a yellow color head, enemies are grey color ghosts and grey color gargoyles with a red torch. In the left images, the gargoyle behind main character is not visible.

The third evaluated scene is composed of the main character and five enemies, and the rest of the scene is considered as background, see Figure 11. The main character is a kitty with a yellow color head, enemies are grey color ghosts and grey color gargoyles with a red torch. We see two different pairs of rear views, Figure 11 left and Figure 11 right. Figure 11 bottom is zoomed out of Figure 11 top. In Figure 11 left, we cannot appreciate the closest enemy behind the main character, while it is clearly visible in Figure 11 left. Thus, we consider better views than the ones in Figure 11 right.

**Figure 12:**
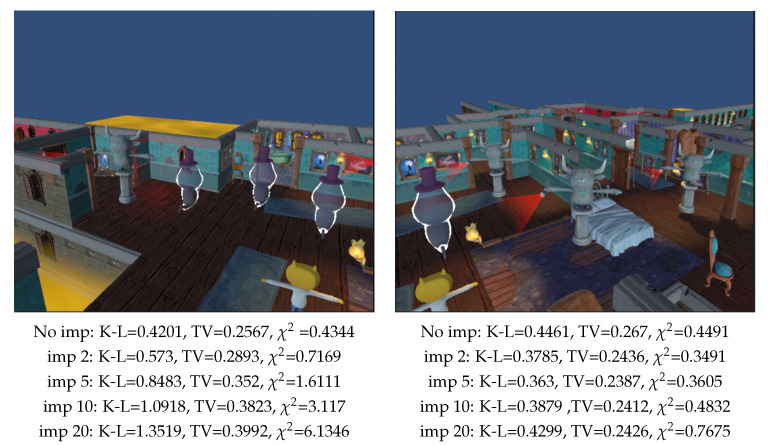
Comparison of measures with importances in a video game (TV: Total Variation, K-L: Kullback–Leibler, and $\chi^{2}$), computed with 100,000 rays. Background area = 50%. Results in top row are given without importance, while in the second, third, fourth, and fifth row importance values of 2, 5, 10 and 20 are, respectively, assigned to gargoyles and importance 1 to ghosts. There are three ghosts and one gargoyle visible in the left image, while three gargoyles and one ghost in the right image.

As another innovative aspect of the implemented method, we consider the importance $\left\{p_{i}\right\}$ of different entities, as described in Section 3.4. Let us envision a scenario in a game with an isometric view positioned behind the main character, featuring a scene populated by eight enemies, four gargoyles and four ghosts. Our objective is to recommend to the player a viewpoint that detects the highest risk. In this case, we deem gargoyles as more offensive than ghosts. Consequently, we assign greater importance to gargoyles. Figure 12 compares two camera views where in Figure 12 left there are three ghosts and one gargoyle, while in Figure 12 right there are three gargoyles and one ghost. Although, at first glance, the view in Figure 12 left might appear preferable due to the enemies being slightly closer, which is reflected in the lower values of the three measures considered, this view contains more ghost-type enemies, which are less aggressive to the player. Therefore, our interest lies in detecting that the most dangerous area is the one in the view in Figure 12 right. However, if we assign greater importance to gargoyles (2, 5, 10 and 20, respectively) while ghosts are given importance 1, we observe that as the gargoyles’ importance increases over ghosts one, K-L and $\chi^{2}$ measures will gradually identify the view in Figure 12 right as superior.

### 4.2. Computation Time

**Figure 13:**
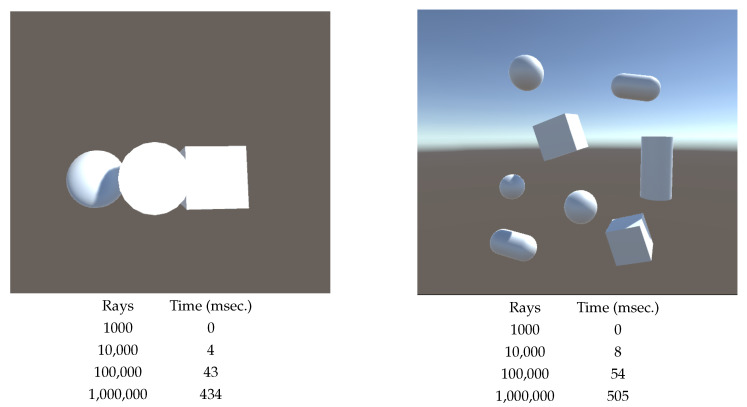
Computing time cost (in msec.) in two scenes and with different numbers of rays. The time corresponds to finding first hit for rays and computing the measures. The computation of areas and generation of rays is conducted only once in a preprocessing step, being the cost for 1,000,000 rays being around half a second.

The computation time, obtained from the Time function provided by Unity, comprises a preprocessing time, where areas are calculated and rays are generated, and the time for finding the first hit for each ray and computing the form factors and the measures. The rays are stored and reused for each viewpoint, as the intersections are computed in camera coordinates. With a PC running Windows 10 Pro 64-bit, equipped with an Intel Core i7-6700K CPU @ 4.00 GHz, 16.0 GB RAM, and an NVIDIA GeForce GTX 1080 graphics card using Unity version 2020.3.30f1, the preprocessing time for 100,000 rays is of the order of half a second. In Figure 13 we show the computation time for finding the first hits and computing the measures for different numbers of rays. Time increases proportionally with the number of rays, as expected. The increased cost in the scene in Figure 13 right with respect to the one in Figure 13 left is due to its increased complexity.

The consistency in results suggests that our implementation is robust and scalable, rendering it suitable for real-time applications and interactive environments.

## 5. Discussion

The Kullback–Leibler divergence is known for its sensitivity to differences in the probability distribution between two sets of data. This makes it especially useful for detecting significant changes in game scenes, as seen for instances in Figure 8, where K-L divergence detects a significant difference between the two scenes while the difference of the values for TV and χ-square divergences and Entropy are within the experimental error threshold. However, K-L divergence can be expensive in terms of calculations in complex scenes.

$\chi^{2}$ metric stands out for its simplicity and cheaper calculation, which makes it suitable for real-time applications. It is useful to detect differences when areas seen are clearly different, such as in Figure 5 and Figure 6. However, it tends to be less sensitive to subtle differences between distributions compared to K-L as shown in Figure 8.

As for the Total Variation, this is a standardized metric that should facilitate the comparison and interpretation of values in different contexts. This metric, as well as the $\chi^{2}$ one and different from the K-L one, takes into account the objects that are not seen from the camera, although the possible advantage of TV and $\chi^{2}$ divergences on K-L divergence is counterbalanced by the use of a background area.

Entropy behaves well in most of our examples, except in Figure 5 upper left, where zooming in on an object increases the entropy and Figure 8 where no improvements are detected. Remember too that the entropy measure considers all objects of equal area and thus the best measure (maximum entropy) would be to see all of them (including background) with an equal form factor, independent of their relative area. On the other hand, divergence measures have the advantage of taking into account the area of the object, and the flexibility of being able to assign importance to them.

All in all, if computation is not an issue and discrimination between measures is a must, K-L divergence can be recommended. If standardization is a must TV divergence can be used, taking care of zooming in on objects. $\chi^{2}$ divergence represents a balance between good discrimination and cheap computation.

## 6. Conclusions and Future Work

We have presented in this paper a framework for camera selection in video games that uses information theoretic f-divergences to give the correlation between the visibility from the camera and the objective or target distribution. The visibility is measured by differential area-to-area form factors that are efficiently computed by casting rays using importance sampling Monte Carlo integration. The target by default is the area of the objects but can be modified by assigning importance to them. Thus our approach allows us to take into account the relative importance and preferences of each element in the game. For instance, we can assign higher weights to main characters or key objects to assure higher visual attention for them, in the function of the scene, player characteristics, and game objectives. This can improve the aesthetics of the game and player immersion and experience. The results show the correctness of our approach and seem to favor the K-L divergence as the most discriminating one. We have also shown that the Shannon viewpoint entropy measure is a particular case of K-L divergence when importances are proportional to the inverse of the area.

Currently, our method does not take into account colors or textures for selecting the best view. As part of future work, we plan to include color, illumination and textures, using for instance the importance mechanism. We will also consider the inclusion of the narrative of the game in the camera selection process, as well as more complex game environments, with several kinds of objects and levels of complexity, and evaluate the impact in the improvement of user experience in the different environments.

Another line of work will be to improve the computation time of measures. Using coroutines, or leveraging Unity’s job system with the burst compiler to parallelize tasks such as raycasting, can significantly improve performance and responsiveness in complex game environments. Coroutines allow heavy computations to be spread over multiple frames, which reduces frame rate drops and maintains smooth gameplay. Furthermore, the job system and the burst compiler offer a more structured and efficient approach to parallel computing, taking full advantage of multicore processors. Improved computational efficiency is important as we plan to integrate more complex physical properties and narrative elements into the camera selection process, ensuring that these advanced features enhance the gameplay experience.

Reinforcement learning combined with a multi-agent system will be considered too. A machine-learning agent would interact with the game environment, making decisions on camera position and obtaining rewards according to improved visual quality. This approach could contribute to a more customized and narrative-oriented camera selection.

We will also investigate the weighted combination of measures and dynamically adjust the weights according to the kind of objects in the game. This approach could benefit from a multi-agent system, that according to the kind of objects (foes, key elements in the plot or main characters) computes the optimal weights for each metric.

Finally, an extension to 2d games will be considered [46].

## Figures and Tables

**Table 1:** Form factor computation validation, see Figure 2. The frustum covers the whole hemisphere. The expected error $\sqrt{F_{i}\left(1-F_{i}\right)/N_{rays}}$ when form-factor = 0.2 and for 10,000 rays is 0.004, for 100,000 rays is 0.00126. For form-factor = 0.1, for 10,000 rays is 0.003, for 100,000 rays is 0.000948.

Values: R=0.5, D=2, k=4. Analytical Form-Factor = **0.2**				
**Total Rays**	**Hit Disc Rays**	**Form-Factor**	**Abs. Error**	**Expected Error**
10,000	2017	0.2017	0.0017	0.004
10,000	2024	0.2024	0.0024	0.004
10,000	2008	0.2008	0.0008	0.004
100,000	19,771	0.19771	0.00229	0.00126
100,000	19,825	0.19825	0.00175	0.00126
100,000	19,835	0.19835	0.00165	0.00126
Values: R=1, D=3, k=3. Analytical form-factor = **0.1**				
Total rays	Hit disc rays	Form-factor	Abs. Error	Expected error
10,000	975	0.0975	0.0025	0.003
10,000	982	0.0982	0.0018	0.003
10,000	1020	0.1020	0.0020	0.003
100,000	9736	0.09736	0.00264	0.000948
100,000	9851	0.09851	0.00149	0.000948
100,000	9925	0.09925	0.00075	0.000948

**Table 2:** Values of the measures for Figure 3 for different number of rays.

Total Rays	K-L	TV	$\chi^{2}$
10,000	0.3466502	0.331814	0.4405105
10,000	0.3530622	0.3346139	0.4405105
10,000	0.2731511	0.3346139	0.3538287
10,000	0.2862652	0.304014	0.369765
10,000	0.3157122	0.3179139	0.4044647
100,000	0.3179567	0.3190739	0.4072793
100,000	0.3164447	0.318334	0.4054418
100,000	0.3170037	0.318584	0.4060861
100,000	0.321581	0.320724	0.4115101
100,000	0.3169065	0.318514	0.4059337

## Data Availability

The original contributions presented in the study are included in the article/supplementary material, further inquiries can be directed to the corresponding authors.

## Appendix A. Monte Carlo Computation of Form-Factors

Suppose first, the frustum covers the whole hemisphere. To solve the form-factor hemisphere integral by Monte Carlo integration with importance sampling [7,8,42], let us first consider that ω direction is given by the pair (θ,ϕ), and that dω=sinθdθdϕ. Then considering as pdf $f\left(\theta ,\phi ,x\right)=\frac{\cos\theta \sin\phi }{\pi }$, and taking *N* random directions ${\left\{\omega_{k}=\left(\theta_{k},\phi_{k}\right)\right\}}_{k=1}^{N}$ according to $\left(\theta ,\phi \right)=\left(\arccos{\sqrt{R}}_{1},2\pi R_{2}\right)$, where $R_{1}$ and $R_{2}$ are random values from a uniform distribution in the interval [0,1], the value of $F(dA_{x},o_{i})$ can be estimated as

$$\begin{array}{ccc} F(dA_{x},o_{i}) & \approx & \frac{1}{N}\sum_{k=1}^{N}\left(\frac{1}{\pi }\right)\frac{v\left(x,o_{i}\left(\omega_{k}\right)\right)\cos\theta_{k}\sin\theta_{k}}{f(\theta_{k},\phi_{k},x_{k})}\\ & = & \frac{1}{N}\sum_{k=1}^{N}\left(\frac{1}{\pi }\right)\frac{v\left(x,o_{i}\left(\omega_{k}\right)\right)\cos\theta_{k}\sin\theta_{k}}{\frac{\cos\theta_{k}\sin\theta_{k}}{\pi }}\\ & = & \frac{1}{N}\sum_{k=1}^{N}v\left(x,o_{i}\left(\omega_{k}\right)\right)=\frac{N_{i}}{N}, \end{array}$$

where $v(x,o_{i}\left(\omega_{k}\right))$ is a boolean that tells us whether object *i* is visible from *x* in direction $\omega_{k}=\left(\theta_{k},\phi_{k}\right)$ and $N_{i}$ is the number of hits on object *i*.

Consider now that we restrict the counting of hits on object $o_{i}$ to the ones that also hit frustum $\Omega_{fr}$. $N_{i}$ is now the number of rays that hit the frustum and object $o_{i}$ and $N_{fr}$ is the total number of rays out of the original *N* cast that hit the frustum. As we only count the first hit, $\sum_{i}N_{i}=N_{fr}$. The form-factors $F(dA_{x},o_{i})$ have to be normalized, thus

$$F\left(dA_{x},o_{i}\right)\approx \frac{N_{i}}{N_{fr}}.$$
